# *Cheleionwatanabei* sp. n., a new species of Stereomerini (Coleoptera, Scarabaeidae, Aphodiinae), and description of the male of *C.jendeki*

**DOI:** 10.3897/zookeys.824.31627

**Published:** 2019-02-12

**Authors:** Showtaro Kakizoe, Munetoshi Maruyama, Kimio Masumoto

**Affiliations:** 1 Entomological Laboratory, Graduate School of Bioresource and Bioenvironmental Sciences, Kyushu University, Motooka 744, Nishi-ku, Fukuoka, 819-0395, Japan; 2 Research Fellow of Japan Society for the Promotion of Science (DC1), Tokyo, Japan; 3 The Kyushu University Museum, Hakozaki 6-10-1, Higashi-ku, Fukuoka, 812-8581, Japan; 4 Kamezawa 3-chôme 14-13-1001, Sumida-ku, Tokyo, 130-0014, Japan

**Keywords:** Aphodiines, new species, Oriental region, Peninsular Malaysia, Southeast Asia, taxonomy

## Abstract

*Cheleionwatanabei***sp. n.** is described from Pahang, Peninsular Malaysia and represents the third species of the genus *Cheleion* Vårdal & Forshage, 2010 (Coleoptera, Scarabaeidae, Aphodiinae, Stereomerini). A description of the previously unknown male of *C.jendeki* Král & Hájek, 2015, and a key to the species of the genus *Cheleion* are also provided.

## Introduction

The aphodiine tribe Stereomerini was established by Howden and Storey (1992) based on the genus *Stereomera* Arrow, 1905, from Singapore. Currently, 9 genera and 22 species from the Oriental and Australian regions are recognized in Stereomerini (Howden and Storey 1992, 2000; Bordat and Howden 1995; Storey and Howden 1996; Maruyama 2009; Vårdal and Forshage 2010; Maruyama and Nomura 2011; Král and Hájek 2015). The Oriental species are known to be extremely rare; all of them are known from single or a few specimens collected so far. Therefore, the males are rare and consequently most entomologists have avoided dissection of the genitalia; male genitalia of Stereomerini have been described only for two Australian species (Howden and Storey 1992; Storey and Howden 1996). Recently we examined two Stereomerini specimens from Peninsular Malaysia: one was found to be a female of an undescribed species belonging to the genus *Cheleion* and the other was identified as a male of *Cheleionjendeki* Král & Hájek, 2015. The description of the new species and the previously unknown male of *C.jendeki* as well as a key to the species of *Cheleion* are provided herein.

## Materials and methods

**Figures 1–4.**
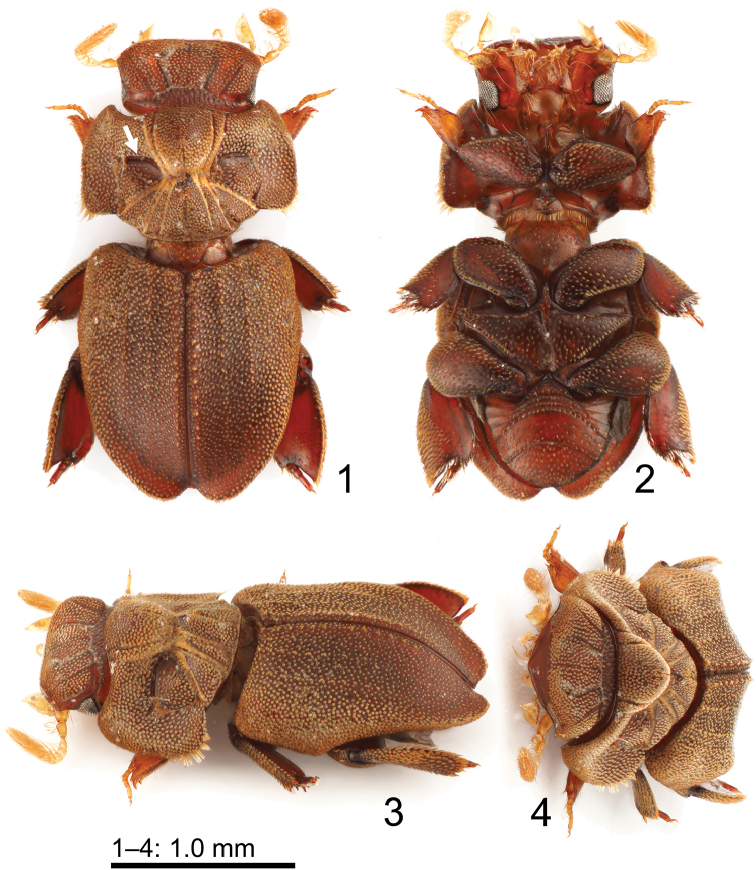
Habitus of *Cheleionwatanabei* sp. n. (♀ holotype) **1** dorsal view (anterior ridge of pronotal depression arrowed) **2** ventral view **3** dorsolateral view **4** anterodorsal view.

**Figures 5–7.**
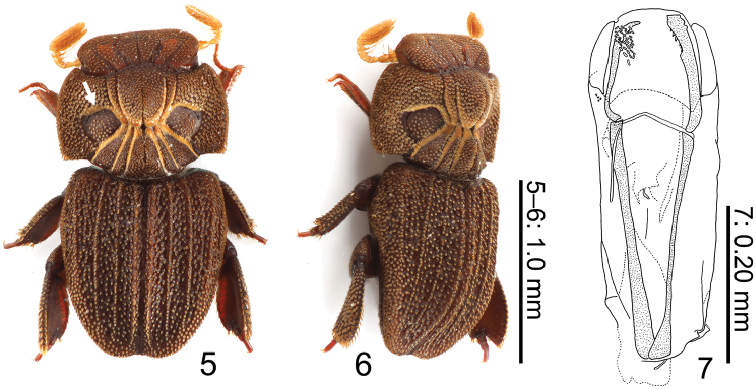
Habitus of *Cheleionjendeki* (♂) **5** dorsal view (anterior ridge of pronotal depression arrowed) **6** dorsolateral view **7** Aedeagus in dorsal view.

**Figures 8–11.**
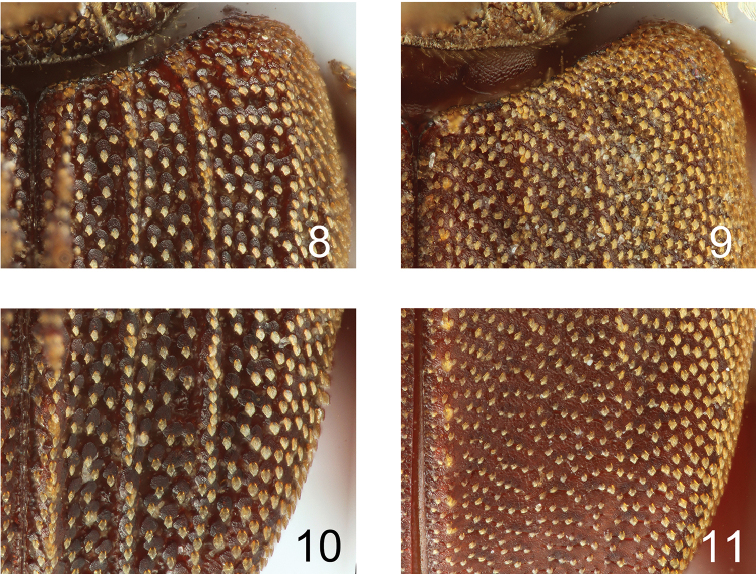
Comparison of scale between *Cheleionjendeki* and *C.watanabei* sp. n. **8, 10***C.jendeki* (♂) **9, 11***C.watanabei* sp. n. (♀ holotype) **8, 9** base of right elytron **10, 11** posterior part of right elytron.

All specimens were dried and mounted on paper cards for morphological observation; dissected genitalia were mounted in Euparal on a small glass plate (10×5 mm), and subsequently glued onto a paper card (6×5 mm) and pinned under the specimen (Maruyama 2004). Specimen photographs were taken with an Olympus OM-D E-M1 Mark II with a Canon MP-E 65 mm 1–5× macro lens and KIPON EF-MFT AF adapter for Figures 1–6, with a Canon EOS 7D Mark II with a Mitutoyo M Plan Apo 20× and a Raynox DCR-150 for Figures 8–11, and subsequently stacked using CombineZP software. Images were edited using Adobe Photoshop CS6. Terminology of the species description follows Howden and Storey (1992), Vårdal and Forshage (2010) and Král and Hájek (2015).

## Taxonomy

### Cheleion watanabei

Taxon classificationAnimaliaColeopteraScarabaeidae

Kakizoe, Maruyama & Masumoto sp. n.

http://zoobank.org/9186941F-6E3F-4EB8-829D-78AB1D4C594F

Figs 1–4 , 9 , 11

#### Type material.

Holotype, female, deposited in the National Museum of Nature and Science, Tsukuba, Japan: “PENINSULAR MALAYSIA, Pahang, Cameron Highlands, Tanah Rata, 8–12. III. 2006, T. Watanabe leg., by FIT [= flight interception trap]”.

#### Distribution.

**Figure 12.**
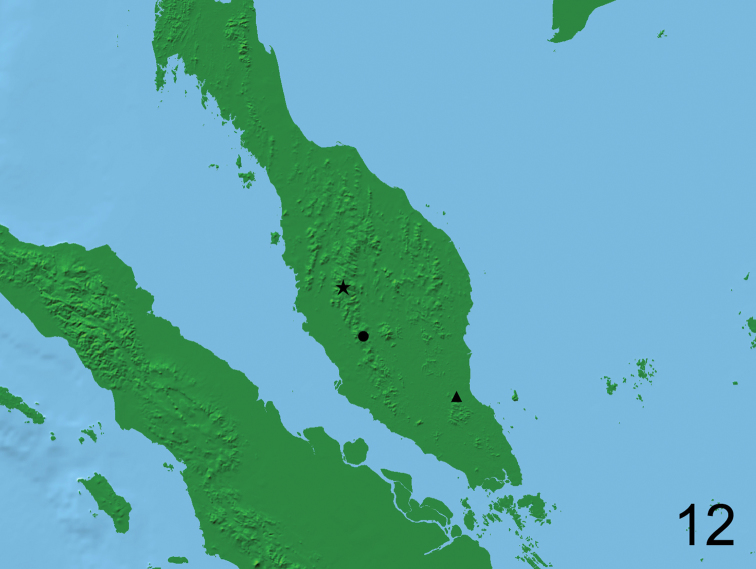
Distribution map of *Cheleion* species: black star *C.watanabei* sp. n., black circle *C.malayanum*, black triangle *C.jendeki*.

(Fig. 12) Peninsular Malaysia, Pahang, Cameron Highlands, Tanah Rata.

#### Etymology.

Dedicated to Mr Takashi Watanabe, the collector of the type specimen.

#### Diagnosis.

This new species is easily distinguished from all known congeners by the following character states: 1) larger body size (total body length ca. 2.5 mm); 2) anterior ridges of the two distinct pronotal depressions short; 3) pads bearing lanceolate scale on the elytra slightly developed; 4) elytral posterior margin strongly depressed; and 5) elytral ridges indistinct.

#### Description.

**Holotype female.** Large species (2.51 mm). Body (Figs 1–4) slightly convex dorsally, dorsal surface more or less covered with appressed lanceolate scales, general color uniformly matte brown. ***Head*** (Figs 1–4) wide, subrectangular in dorsal view, with densely appressed lanceolate scales. Clypeus impunctate, shiny, apically pointed and reflexed under head. Frons flat with five anteriorly divergent furrows, second and fourth furrows strongly curved medially. Genal tip slightly obtusely angular in dorsal view. Eyes large, visible ventrally, less visible dorsally. Occiput with numerous small, longitudinal punctures. Antennae long, amber colored, with long setae; club lamellae long. Maxillary palpi as long as head, amber colored, with securiform ultimate palpomeres. ***Prothorax*** (Figs 1–4) large and transverse, anterior edge slightly bisinuate, side edges weakly rounded in dorsal view. Base of pronotum with a median protrusion, wider than base of elytra. Pronotal disc with seven furrows medially, concave at middle of hourglass pattern, with tufts of dense trichomes, with densely appressed lanceolate scales; mid furrow shallower than lateral furrows. Lateral furrows large, flat. Subtriangular depressions delineated by more furrows sparsely appressed lanceolate scales medially. Anteromedial disc distinctly high, tuberculate; promedial disc and posterolateral sides slightly low, less tuberculate than anteromedial disc. Prosternum fairly elevated and expanded anteriad and posteriad, rugose; anterior part grooved longitudinally and sinuate apically, posterior part hastate; prosternal spine apically pointed. ***Scutellum*** (Figs 1, 3, 4, 9) triangular, notably small. ***Elytra*** (Figs 1–4, 9, 11) approximately as wide as pronotum and only slightly longer than pronotum and head combined; feebly tapered posteriad, fairly rounded apically; posterior margin strongly depressed. Each elytron with three indistinct ridges; intervals (between ridges) flat, rugose, with densely appressed lanceolate scales. Epipleura broadly inflexed; posterior two-thirds of lateral edge slightly recurved to allow free movement of metalegs. Macropterous. ***Mesoventrite*** (Fig. 2) strongly narrow with alutaceous and punctured surface. ***Metaventrite*** (Fig. 2) flat, alutaceous, subtriangular, tapering, widest anteriorly, grooved along midline, with coarse, macrosetigerous punctures. ***Legs*** (Figs 1–4) short with broad femora and tibiae. Femora shiny, covered with coarse, macrosetigerous punctures. Protibiae flattened; each with two teeth on outer edge, sparsely macrosetose outward. Protarsi pentamerous, amber colored, long, sparsely macrosetose medially, inserted well before protibial apex. Proclaws normal, symmetrical. Meso- and metatibiae with concave, sparsely macrosetose apex; each with five inconspicuous terminal spurs; dorsal sides shiny, glabrous; ventrolateral sides with densely appressed lanceolate scales. Meso- and metatarsi tetramerous, short, compacted-complanate, sparsely macrosetose medially. Meso- and metaclaws slightly weak, symmetrical. ***Abdomen*** (Fig. 2) with five visible ventrites apparently fused, covered with coarse, dense, macrosetigerous punctures. Pygidium exposed, strongly punctate proximally, less strongly apically.

**Male.** Unknown.

#### Measurements.

Body length 2.51 mm; maximum width of head 0.79 mm; median dorsal length of pronotum 0.74 mm, maximum width 1.16 mm; sutural length of elytra 1.15 mm, maximum length 1.26 mm, maximum width 1.22 mm.

### Cheleion jendeki

Taxon classificationAnimaliaColeopteraScarabaeidae

Král & Hájek, 2015

Figs 5–7 , 8 , 10

Cheleion jendeki Král & Hájek, 2015: 88 (original description based on a female).

#### Material examined.

PENINSULAR MALAYSIA, Pahang, near the gate of Taman Negeri Endau Rompin, alt. 30 m, 8–23. III. 2015, S. Kakizoe, K. Hoshino, S. Kakinuma & H. Osaki leg., by FIT, 1 ex. male.

#### Additional description based on male.

***Legs*** (Figs 5, 6). Protarsi pentamerous, amber colored, long, sparsely macrosetose medially, inserted well before protibial apex. ***Aedeagus*** (Fig. 7). Symmetrical. Phallobase elongate, cylindrical. Parameres short, almost a quarter length of basal piece. Phallus a little longer than parameres, rounded apically. Struts long, almost two-thirds length of tegmen.

#### Sexual dimorphism.

No sexual dimorphism detected.

#### Measurements.

Body length 1.80 mm; maximum width of head 0.67 mm; median dorsal length of pronotum 0.61 mm, maximum width 0.94 mm; sutural length of elytra 0.92 mm, maximum length 0.96 mm, maximum width 0.92 mm.

#### Remarks.

No males were known for the genus *Cheleion* so far, therefore a description of the male of *C.jendeki* is provided here. Furthermore, Král and Hájek (2015) reported that the tarsal formula for *C.jendeki* was 4-4-4, but it is actually 5-4-4. Therefore, the tarsal formula of *C.malayanum* given by Vårdal and Forshage (2010) is probably also inaccurate.

### Key to the species of the genus *Cheleion*

**Table:** 

1	Large (2.5 mm); anterior ridges of two distinct pronotal depressions short (Fig. 1, arrowed); elytral ridges indistinct, each pad bearing slightly-developed lanceolate scales, densely appressed on elytra; elytral posterior margin strongly depressed	***C.watanabei* sp. n.**
–	Small (1.8–1.9 mm); anterior ridges of distinct pronotal depressions long (Fig. 5, arrowed); elytral ridges distinct, each pad bearing well-developed lanceolate scales, moderately appressed on elytra, elytral posterior margin not or feebly depressed	**2**
2	1^st^ and 5^th^ divergent furrows on head weakly s-shaped; distinct pronotal depressions small, subtriangular; prosternal spine apically pointed; elytral ridges broad, most pads bearing lanceolate scale confluent to subconfluent	***C.malayanum* Vårdal & Forshage, 2010**
–	1^st^ and 5^th^ divergent furrows on head straight; distinct pronotal depressions large, subrectangular; prosternal spine apically not pointed; elytral ridges narrow, most pads bearing lanceolate scale separated	***C.jendeki* Král & Hájek, 2015**

## Supplementary Material

XML Treatment for Cheleion watanabei

XML Treatment for Cheleion jendeki
